# Laminin 411 acts as a potent inducer of umbilical cord mesenchymal stem cell differentiation into insulin-producing cells

**DOI:** 10.1186/1479-5876-12-135

**Published:** 2014-05-20

**Authors:** Huiting Qu, Xiaoli Liu, Yihong Ni, Yang Jiang, Xiaoli Feng, Juan Xiao, Yanan Guo, Dexiao Kong, Ai Li, Xiaomei Li, Xianghua Zhuang, Zhilun Wang, Yongjing Wang, Yali Chang, Shihong Chen, Feng Kong, Xuhua Zhang, Shengtian Zhao, Yi Sun, Dawei Xu, Daoqing Wang, Chengyun Zheng

**Affiliations:** 1 Departments of Hematology and Cellular Therapy, the Second Hospital of Shandong University, Jinan, Shandong, PR China; 2 Institute of Biotherapy for Hematological Malignancies, Shandong University, 247 Beiyuan Road, Jinan, Shandong, PR China; 3 Department of Endocrine, the Second Hospital of Shandong University, Jinan, Shandong, PR China; 4 Clinical Laboratory, the Second Hospital of Shandong University, Jinan, Shandong, PR China; 5 Department of Oncology, the Second Hospital of Shandong University, Jinan, Shandong, PR China; 6 Department of Hematology, Jinan Central Hospital Affiliated to Shandong University, Jinan, Shandong, PR China; 7 Central Lab, the Second Hospital of Shandong University, Jinan, Shandong, PR China; 8 Department of Urology, the Second Hospital of Shandong University, Jinan, Shandong, PR China; 9 BioLamina AB, Sundbyberg, Stockholm, Sweden; 10 Center for Molecular Medicine, Division of Hematology, Karolinska Institutet, Stockholm, Sweden

**Keywords:** Laminin 411, Mesenchymal stem cell, Insulin-producing cell

## Abstract

**Background:**

Diabetes mellitus (DM) is an incurable metabolic disease constituting a major threat to human health. Insulin-producing cells (IPCs) differentiated from mesenchymal stem cells (MSCs) hold great promise in the treatment of DM. The development of an efficient IPC induction system is a crucial step for the clinical application of IPCs for DM. Laminin 411 is a key component of the basement membrane and is involved in the regulation of cell differentiation; however, little is known about a role of laminin 411 in the regulation of IPC differentiation from human MSCs.

**Methods:**

MSCs were isolated from human umbilical cord (UC-MSCs) and expanded in an *in vitro* culture system. UC-MSCs were then cultured in the IPC induction and differentiation medium in the presence of laminin 411. Flow cytometry, Quantitative realtime PCR, immunofluorescence staining, ELISA, Western blotting and other techniques were applied to determine IPC generation, insulin expression and related mechanisms. To evaluate potential therapeutic efficacy of IPCs induced from UC-MSCs, a type-1 diabetes (T1DM) rat model was generated using streptozotocin. Blood glucose, insulin levels, and survival of rats were monitored periodically following intravenous injection of the tested cells.

**Results:**

Laminin 411 markedly induced the expression of the genes *Foxa2* and *Sox17*, markers for pancreatic precursor cells, efficiently induced IPC differentiation from MSCs, and up-regulated insulin expression at both mRNA and protein levels. Furthermore, the expression of the genes known to govern insulin expression including *Pdx1* and *Ngn3* was markedly induced by laminin 411, which suggests that *Pdx*1 and *Ngn*3 signaling pathways are involved in laminin 411 induced-insulin expression machinery. More importantly, administration of laminin 411-induced IPCs rapidly and significantly down-regulated fasting blood glucose levels, significantly reduced the HbA1c concentration and markedly improved the symptoms and survival of T1DM rats.

**Conclusions:**

Our results demonstrate that laminin 411 acts as a potent differentiation inducer of IPCs from UC-MSCs via the *Pdx1* and *Ngn3* signaling pathways. Moreover, transfusion of laminin 411 induced-IPCs more efficiently improves symptoms and survival of T1DM rats. These novel finding highlights a potential clinical application of laminin 411 induced-IPCs in the treatment of T1DM, which calls for further studies.

## Introduction

Diabetes mellitus (DM) is a group of metabolic disorders of carbohydrate metabolism, which constitutes a major threat to human health worldwide, particularly in industrialized countries. The main feature of the disease is hyperglycemia resulting from overproduction and underutilization of glucose [1]. DM is briefly classified into two subtypes, type-1 (T1DM) and type-2 (T2DM). Classical treatments of DM consist of insulin injection and orally taking hypoglycemic drugs. Such daily therapeutic approaches markedly affect life quality of DM patients due to side-effects of the hypoglycemic drugs and suffering of injection. Thus, novel treatments with long-lasting efficacy and lower side-effects are urgently needed. Studies on islet transplantation for DM have shown promising results [2,3], but the long-term efficacy is still unsatisfying. In addition, shortage of donors for islets and poor *in vitro* expansion capacity of islets remain unresolved, which limit the use of such therapy in the clinical setting. Therefore, developing alternative cellular therapy strategies for DM is an urgent task.

Mesenchymal stem cells (MSCs) have gained interest because of its potential application in regeneration medicine and cytotherapy. MSCs are a kind of pluripotent adult stem cells with the advantage of lower immunogenicity and the ability toward osteogenesis, adipogenesis, and chondrogenesis differentiation [4]. MSCs can be obtained from numerous sources, such as bone marrow (BM), umbilical cord tissue, adipose tissue, dental pulp, amniotic fluid, and pancreatic islets [5]. Previous studies demonstrated that intravenous infusion of MSCs decreased blood glucose level in Balb/c diabetes mice [6], and prevented autoimmune diabetes in NOD mice [7]. In contrast to the results obtained from the above animal studies, our polite clinical trial using intravenous transfusion of human umbilical cord Wharton’s jelly derived MSCs (UC-MSC) did not yield satisfying results (unpublished data).

Tang et al. reported that BM-derived stem cells derived from Balb/c mice could be trans-differentiated into insulin-producing cells (IPCs), but this process requires more than 2 months to generate the insulin-producing cluster [8]. In 2007, Karnieli et al described for the first time a protocol for human BM-derived MSCs differentiated into IPCs by gene manipulation [9]. Since then, various protocols for IPC differentiation have been published. Briefly these protocols can be divided into 2 types: one is to apply gene manipulation techniques (e.g. *Pdx1* transfection) [10], and another is to use small molecules and/or growth factors to induce IPC differentiation. Conceivably, gene manipulation requires the technique transfecting target cells using a viral vector encoding specific genes. For the concern of bio-safety, the cells having undergone gene manipulation are not very promising for clinical use. Non-gene manipulation protocols for IPC differentiation has been described, however, their induction efficiency of IPC differentiation from stem cells remains poor, only 10-20% differentiation rate [11]. Thus, it is urgently demanding a highly efficient protocol for IPC generation.

Laminin is a heterotrimer glycoprotein that contains alpha, beta, and gamma chains. To date, at least 19 laminin isoforms have been identified [12], and it is named according to its sub-chain types, e.g., laminin 411 comprises the α4, β1, and γ1 chains [13]. Laminin is a key component of the basement membrane, and is involved in the structural scaffold, cell proliferation, and differentiation. Previous studies showed that laminin 111 promoted the differentiation of fetal mouse pancreatic beta cells [14], and induced the expression of islet cell markers in the hepatic oval cells *in vitro*[15]. Moreover, Laminin was observed to promote differentiation of the *hTERT*-over-expressed human BM derived-MSCs into IPCs [16].

In this study, we tested potential activity of laminin 411 on induction of human UC-MSC into IPCs without any gene manipulation and therapeutic effects of the IPCs on DM animal models. Our results demonstrated that the laminin 411-based protocol significantly enhanced the induction of IPC differentiation from UC-MSC, and these IPCs produced significantly higher levels of insulin than those without laminin 411. Moreover, the animal study showed that intravenous administration of laminin 411-induced IPCs (IPC + 411) rapidly reduced fasting blood glucose (FBG) levels, significantly prolonged the survival and improved DM signs when compared to untreated controls.

## Materials and methods

### UC-MSC culture and expansion

Human umbilical cord (UC) specimens were collected from the obstetrical department of the Second Hospital of Shandong University. Ethic committee of Second Hospital of Shandong University approved the study and informed consents were obtained from all donors. UC tissue we rinsed with phosphate-buffered saline (PBS). Wharton’s jelly tissue was chopped into small pieces and digested with collagenase and trypsin (Gibco BRL, Gaithersburg, MD, USA). Finally, UC-MSCs were cultured in DMEM complete medium (Thermo, Logan, UT, USA), containing 10% defined fetal bovine serum (Biowest, NuaillÃ, France), L-glutamine (2 mM; Life Technologies, Carlsbad, CA, USA), growth factors (R&D Systems, Lille, France), penicillin and streptomycin (Life Technologies). Cells were cultured in a humidified atmosphere with 5% CO_2_ at 37°C. After 72 hrs of incubation, non-adherent cells were carefully removed and fresh medium were added. When the cell density reached 80% confluence, they were passaged.

### UC-MSC immunophenotyping

The uniform spindle-fibroblastic morphology and adherent phenotype was identified under an inverted microscope (Nikon Corp., Tokyo, Japan). UC-MSCs were resuspended in PBS, stained with fluorescence-labeled monoclonal antibodies CD34, HLA-DR, CD105, CD73, CD90, and CD45 (BD, Franklin Lakes, NY, USA), and then incubated for 30 min at 4°C in the dark. Appropriate isotype controls were included (for each antibody isotype). The expression of the above cell surface markers was evaluated using LSRFortessa Flow Cytometer (BD).

### UC-MSC differentiation assays

Differentiation kits (Life Technologies) were used according to the manufacturer’s instructions. Briefly, UC-MSCs were seeded at 1 × 10^4^/cm^2^, and incubated in the adipose and osteoblast differentiation culture media for 21 d, respectively. Adipocytes and osteocytes were stained with oil red O and Alzarin red, respectively. For chondrogenesis differentiation, UC-MSCs were placed in a DMEM complete culture media at 1.6 × 10^7^ cells/ml. UC-MSCs were seeded with 5 μl droplets placed in chondrogenesis culture media for 14 d. The micromass cultures were then stained with Alcian blue.

### IPC differentiation protocol

**Figure 1:**
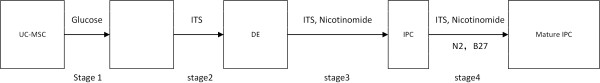
**Illustrations of the IPC differentiation protocol.** Undifferentiated cells were suspended in complete culture media and aliquots of 2.5 × 10^5^ cells and placed in 6-well plates overnight. The medium was replaced with complete culture media either with (control) or without pre-coated 5 μg/mL laminin 411 and 25 mM glucose for 3 d (stage I). At stage II, the media were refreshed with DMEM/F-12 medium containing Insulin-Transferin-Selenium-A for another 4 d. At stage III, 10 mM nicotinamide was added to the media, and culture lasted for 3 days. At stage IV, the medium was changed with the same supplements at stage III, but N2 and B27 supplements were added and incubated for 4 d.

UC-MSCs from passage 4 were used for IPC differentiation (Figure 1). Undifferentiated cells were suspended in complete culture media and aliquots of 2.5 × 10^5^ cells, and placed in 6-well plates overnight. The medium was replaced with complete culture media either with or without pre-coated 5 μg/mL laminin 411 (Biolamina, Stockholm, Sweden) and 25 mM glucose for 3 d (stage I). At stage II, the media were refreshed with DMEM/F-12 medium containing Insulin-Transferin-Selenium-A (ITS-A, BD) for another 4 d. At stage III, 10 mM nicotinamide (MP, USA) was added into the media, and the culture lasted for 3 days. At stage IV, the medium was changed with the same supplements as at stage III, but N2 and B27 supplements (Invitrogen) were added and incubated for 4 d.

### Dithizone (DTZ) staining

DTZ (Sigma-Aldrich, St. Louis, MO, USA) stock solution was prepared by dissolving 100 mg of DTZ in 5 ml DMSO. The induced cells were added into 1 mL 1× PBS buffer and 10 μL DTZ stock solution, and then incubated at 37°C for 15 min. The crimson red stained clusters were examined under a phase-contrast microscope (Nikon Corp.).

### Immunofluorescence

Cultured cells and clusters were fixed, permeabilized in PBS +0.1% Triton X-100, and then blocked for 30 min in PBS +0.5% Tween-20 (PBST) containing 1% BSA. Cells were incubated with insulin and *Pdx1* primary antibodies, followed by the incubation with the secondary antibodies. Cell nuclei were stained with DAPI (Life Technologies). Images were acquired with fluorescence microscope (Nikon Corp.). The mean fluorescence density of the Pdx1 positive staining area was quantified by ImageJ software (NIH, USA).

### Quantitative real time PCR (qPCR)

Total RNA was extracted using Trizol reagent (Life Technologies) following the manufacturer’s instructions. Up to 5 μg total RNA were subjected to reverse transcription into cDNA with M-MLV enzyme (Life Technologies) at 37°C for 50 mins in the presence of an oligo-dT primer. qPCR was performed using Master cycler Realplex 2 (Eppendorf, Hauppauge, NY, USA). The primer sequences were listed in Table 1. The quantification of target gene expression was calculated using Eppendorf realplex software (Eppendorf).

**Table 1:** Nucleotide sequence of the primers used in this study

	**Gene**	**Sequence (5′-3′)**
1	Pdx1 F	GGAGCCGGAGGAGAACAAG
	Pdx1 R	CTCGGTCAAGTTCAACATGACAG
2	Pax4 F	CAGCGCTGCTGGACTT
	Pax4 R	CAGCGCTGCTGGACTT
3	Insulin F	ACCAGCATCTGCTCCCTCTA
	Insulin R	GGTTCAAGGGCTTTATTCCA
4	Ngn3 F	CTATTCTTTTGCGCCGGTAGA
	Ngn3 R	CTCACGGGTCACTTGGACAGT
5	Glucagon F	CCCAAGATTTTGTGCAGTGGTT
	Glucagon R	CAGCATGTCTCTCAAATTCATCGT
6	Foxa2 F	CTGAGCGAGATCTACCAGTGGA
	Foxa2 R	CAGTCGTTGAAGGAGAGCGAGT
7	Sox17 F	GCATGACTCCGGTGTGAATCT
	Sox17 R	TCACACGTCAGGATAGTTGCAGT
8	GAPDH F	GCACCGTCAAGGCTGAGAAC
	GAPDH R	TGGTGAAGACGCCAGTGGA

F: forward primer; R: reverse primer.

### Western blot

Cell lysates were extracted using protein extraction reagent RIPA buffer containing Triton-x-100 (Sigma-Aldrich) and a protease inhibitor cocktail (Roche Applied Sience, Indinapolis, IN, USA). Samples were loaded onto a 10% SDS-polyacrylamide gel. Proteins were transferred onto PVDF membrane (Millipore, Billerica, USA) with Trans-Blot® SD Semi-Dry Electrophoretic Transfer Cell (Biorad, Marnes-La-Coquette, France). The membrane was incubated overnight at 4°C with primary antibodies, *Pdx1* (Cell Signaling Technology, Beverly, MA), *Ngn3* (Cell Signaling Technology), and GAPDH (Cell Signaling Technology). After washing, the membrane was incubated with horseradish peroxidase-conjugated goat anti-rabbit IgG (Santa Cruz, Dallas, Texas, USA) or anti-mouse IgG (Santa Cruz). The membrane was washed and developed using a chemiluminescent HRP substrate (Millipore). Several exposure times were tested for both ECL with X-ray film and ECL with CCD imager.

### Insulin release assay

IPCs at stage IV from each of the groups described above were gently washed twice with PBS. The cells were then pre-incubated in KRB culture media containing glucose (25 mM) at 37°C for 2 hrs and supernatants were then collected for insulin quantification. An ELISA kit (Alpco, Salem, USA) was used to assess insulin concentration according to manufacturer’s instructions. Insulin levels were calculated according to the standard curve.

### Animals

Wistar rats (male, 6 to 8 weeks old) were purchased from the Experimental Animal Centre of Shandong University. Rats were housed in sterile cages under laminar flow hoods in a temperature-controlled room with a 12 h light/dark schedule, and fed with autoclaved chow and water. The experimental protocol and design of the study were in accordance with the institutional guidelines for animal experiments and approved by the institutional Animal Ethics Research committee of the Second Hospital of Shandong University.

### Cell transfusion in rats

Rats were made hyperglycemic by a single i.p. injection of 60 mg streptozotocin (STZ; Sigma–Aldrich) per kg of body weight after fasting [8]. There were 4 rats in each group tested, and the cells were injected through a tail vein. When blood glucose reached levels over 16.7 mol/l within 3 to 7 d of STZ injection, the cells (UC-MSCs, IPCs with or without exposure to laminin 411 and normal saline groups) were transfused via tail veins. Fast blood glucose (FBG) levels were monitored twice a week using Blood Glucose Test Strips (Roche Diagnostics, Basel, Switzerland) and HbA1c levels were quantified using liquid chromatography analyser (Tosoh Europe, Tessenderlo, Belgium).

### Statistical analysis

The Student’s *t* test and one way ANOVA was applied to compare differences between the groups. Data were shown as mean ± SEM. Pearson’s correlation coefficient was used to test for significance in linear relationships between variables. SPSS 20.0 software was used for statistical analysis. *P* < 0.05 was considered as statistical significance.

## Results

### UC-MSC culture and expansion

**Figure 2:**
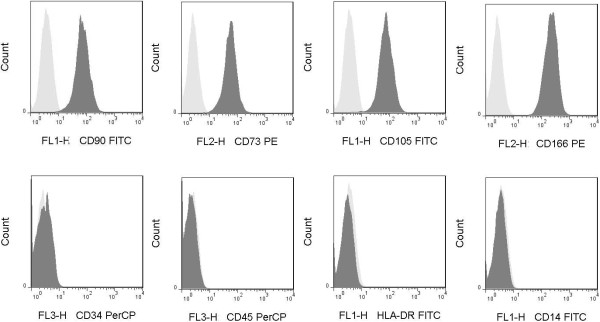
**UC-MSC phenotype analysis.** MSCs derived from umbilical cord were detached with trypsin-EDTA and stained with fluorescence antibodies against surface molecules indicated and analyzed by flow cytometry present in histogram plots. Appropriate isotype controls were performed (for each antibody isotype) to assess cell auto-fluorescence and background staining. MSCs were positive for CD73 (99%), CD90 (99%), CD105 (95%), CD166 (98%), negative for hematopoietic marker CD34 (1.7%), leukocyte common antigen CD45 (2.2%), HLA-DR (1.3%) and monocyte marker CD14 (1.5%).

**Figure 3:**
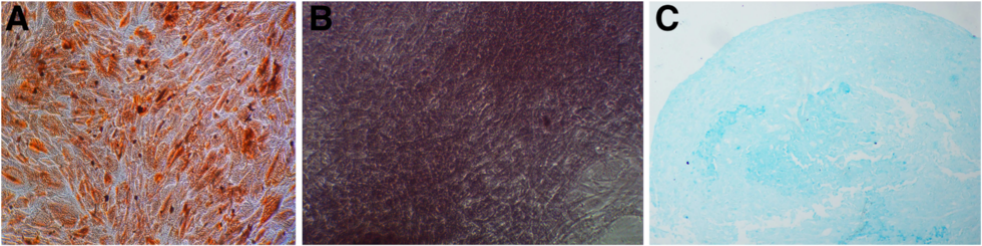
**Evaluation of UC-MSC pluripotency. (A)** P4 UC-MSCs were induced with differentiation kits according to manufacturer’s instructions. After 21 days of adipogenesis differentiation, adipocytes were stained by oil red O. **(B)** After 21 days of osteogenesis differentiation, osteocytes were stained with Alizarin red. **(C)** At the end of chondrogenesis differentiation, chondrocyte mass was stained with Alcian blue. Representatives are shown at 100 × magnification.

UC-MSCs were spindle shaped and adhered to the plastic flask. The phenotype of UC-MSCs was in accordance with the International Society for Cellular Therapy 2006 minimal criteria. UC-MSCs were positive for CD73 (99%), CD90 (99%), CD105 (95%), CD166 (98%), and negative for hematopoietic marker CD34 (1.7%), monocyte marker CD14 (1.5%), leukocyte common antigen CD45 (2.2%), and HLA-DR (1.3%) (Figure 2). To demonstrate differentiation pluripotency, UC-MSCs were subjected to conditions known to induce differentiation into bone, cartilage, and adipose cells. UC-MSCs were induced into adipocyte, osteocyte, and chondrocyte in vitro by oil red O, Alizarin, and Alcian blue, respectively (Figure 3).

### *In vitro* IPC differentiation

**Figure 4:**
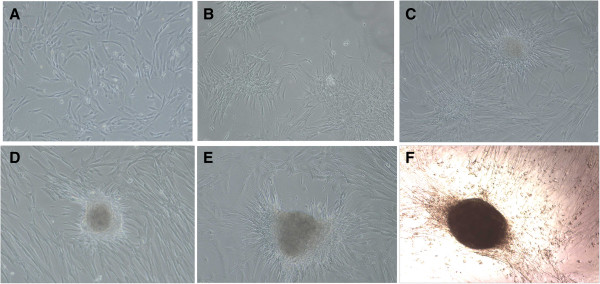
**IPC differentiation. (A)** UC-MSCs culture before IPCs differentiation induction. **(B)** UC-MSCs aggregated together during the differentiation stage 2. **(C)** Insulin clusters were formed at late stage 2. **(D)** IPC clusters at the end of the last stage. **(E)** Adding laminin 411 group formed the IPC clusters. **(F)** DTZ stained the IPCs cluster. Representatives are shown at 100 × magnification.

**Figure 5:**
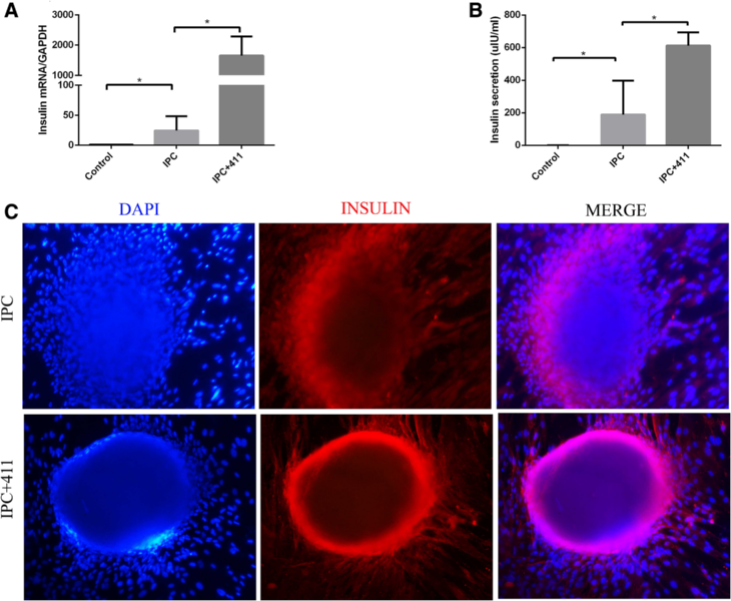
**RNA and protein level of insulin in IPCs. (A)** After glucose stimuli IPCs with or without laminin 411 group mRNA level of insulin was determined using RT-PCR and expressed relative to an undifferentiated control and normalized to GAPDH. **(B)** Insulin release assay. At the end of the 4 stage culture of IPCs, high concentration of glucose was added. Supernatant was collected and tested by ELISA. **(C)** Immunofluorescence staining of insulin of IPCs with or without laminin 411. After culturing them for 4 stages, IPCs with or without laminin 411 were incubated with anti-human insulin antibody. Nuclei were stained blue with DAPI. Each bar is the mean ± SEM (n = 3), *P < 0.05.

We first evaluated UC-MSCs to IPCs differentiation (Figure 4A) in a 4 stage protocol according to previously published protocols with modifications [17,18]. UC-MSCs aggregated together during the differentiation at stage 2 (Figure 4B). At the end of the second stage, islet clusters formed gradually. With continuous incubation, IPC clusters increased in number and mass (Figure 4C and D). IPC clusters were stained with DTZ by the end of the last stage (Figure 4F). RNA was extracted from the IPC clusters, and qPCR) was performed to determine mRNA expression level of insulin and insulin related genes, including *Pdx*1, *Ngn*3 and *Pax*4. *Pdx*1 and insulin mRNA levels of IPCs significantly increased than those in the control group. When stimulated with a high concentration of glucose, the IPCs could secrete insulin, as determined by ELISA (Figure 5B). Collectively, IPCs were successfully induced using a published method, but the insulin secretion was low. Next, we determined whether adding extracellular matrix recombination human laminin 411 would increase the yield of IPCs.

### Laminin 411 increased *Ngn3* and *Pdx1* expression

**Figure 6:**
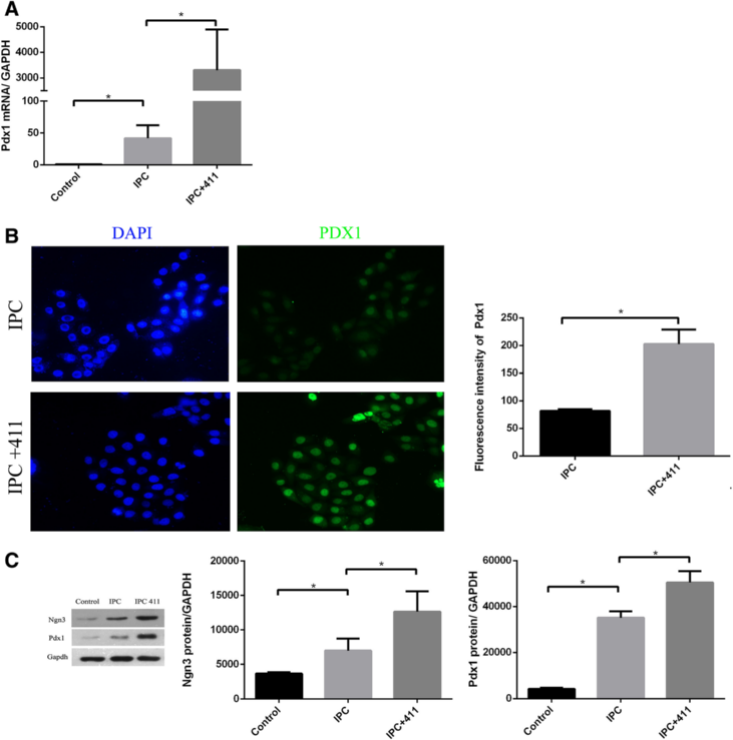
**RNA and protein levels of insulin-related genes in IPCs. (A)** After glucose stimuli IPCs with or without laminin 411 group mRNA level of *Pdx1* was determined using RT-PCR and expressed relative to an undifferentiated control and normalized to GAPDH. **(B)** Immunofluorescence staining of *Pdx1* in IPCs with or without laminin 411. After culturing them for 4 stages, IPCs with or without laminin 411 were incubated with anti-human *Pdx1* antibody. Nuclei were stained blue with DAPI. Quantitative analysis of mean fluorescence density of Pdx1 staining was shown on the left. **(C)** Western blot analysis. Protein level of *Ngn3* and *Pdx1* of IPCs with or without laminin 411. Each bar is the mean ± SEM (n = 3), *P < 0.05.

UC-MSCs were cultured in the differentiation media with and without exposure to laminin 411 for IPC generation and maturation. Our result showed that culture system with laminin 411 yielded more clusters than the one without laminin 411 (24.3 ± 1.0 v.s 14.7 ± 1.8 clusters/well). Moreover, as shown in Figure 4E, the clusters of IPC generated in the system with laminin 411 contained more cells than the one without laminin 411. At the end of the 4 stage culture, total RNA were extracted, and insulin related genes were assessed using qPCR. IPCs induced in the system with laminin 411 (IPC + 411) showed significantly higher *Pdx1* mRNA expression than the one without laminin 411 (IPC) (*P* < 0.05, Figure 6A). Consistently, Pdx1 protein level was significantly up-regulated by laminin 411, as determined by immunofluorescence staining (Figure 6B) and Western blot analysis (Figure 6C). The *Ngn*3 mRNA expression level of the IPCs + 411 was numerically higher than that of the IPCs, but the difference between these two groups did not reach significance (*P* > 0.05). Nevertheless, the protein expression level of *Ngn*3 of the IPCs + 411 was significantly higher than that of the IPCs (*P* < 0.05, Figure 6C). There were no significant difference in the expression of other insulin related genes, *Pax*4, *Pax*6 and *Glucagon*, at mRNA and protein levels between IPC + 411 and IPC (data not shown).

### Laminin 411 augmented insulin expression and secretion

IPC + 411 showed significantly higher insulin mRNA expression than IPC (Figure 5A). After stimulation with a high concentration of glucose for insulin release, the insulin concentration in the supernatants of IPC + 411 was approx. 4 fold higher than that of IPC (612.1 ± 40.78 μIU/ml v.s 189.8 ± 93.05 μIU/ml, *P <* 0.05) (Figure 5B). Insulin expression in the IPC clusters of the two groups was further confirmed by immunofluorescence staining of insulin (Figure 5B).

### Laminin 411 induced differentiation of MSC into pancreatic precursor

**Figure 7:**
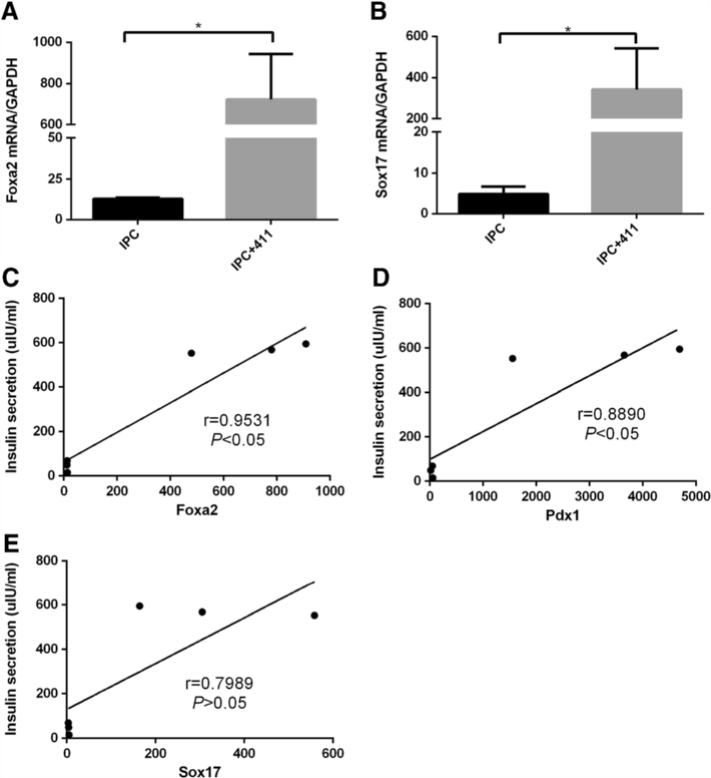
**Definitive endoderm (DE) marker *Sox17 *and *Foxa2 *expression level and correlation analysis. (A)** IPCs with or without laminin 411 group mRNA level of *Foxa2* was determined using RT-PCR and expressed relative to an undifferentiated control and normalized to GAPDH. **(B)** mRNA level of *Sox17* in IPCs with or without laminin 411 was determined using RT-PCR and expressed relative to an undifferentiated control and normalized to GAPDH. **(C)** Correlation of insulin secretion with *Foxa2* gene expression (r = 0.9531, n = 3, *P <* 0.05). **(D)** Correlation of insulin secretion with *Sox17* gene expression (r = 0.7989, n = 3, *P >* 0.05). **(E)** Correlation of insulin secretion with *Pdx1* gene expression (r = 0.8890, n = 3, *P <* 0.05). Bars: mean ± SEM (n = 3), and *P < 0.05.

Li et al. reported that adipose-derived MSC (AD-MSC) could be induced into IPCs through a pancreatic precursor formation pathway [19]. It was further confirmed in an Embryonic Stem Cells (ESCs) differentiation protocol [20]. So we want to know whether laminin 411 was through the same pathway to induce IPC generation. To this end, the cells were harvested from the end of the second stage of differentiation to compare the expression of *Foxa2* and *Sox17*, markers for pancreatic precursor cells [21]. As expected, *Foxa2* and *Sox17 mRNA* expression level of the cells exposed to laminin 411 was significantly higher than that of the ones without exposure to laminin 411 (*P <* 0.05, Figure 7A, 7B), suggesting a role of laminin 411 in the induction of pancreatic precursor differentiation from MSCs.

### Positive correlation between insulin secretion and insulin related genes

Having analyzed the correlation between insulin secretion and insulin related gene expressions, we found that the insulin secretion level was positively correlated with *Pdx1* and *Foxa2* gene expression, respectively (*P <* 0.05 by Spearman’s correlation, Figure 7C, 7D), but not with *Sox17 *(*P >* 0.05 by Spearman’s correlation, Figure 7E).

### IPCs + 411 rapidly and significantly down-regulated glucose, improved survival and reduced HbA1c level in a T1DM animal model

**Figure 8:**
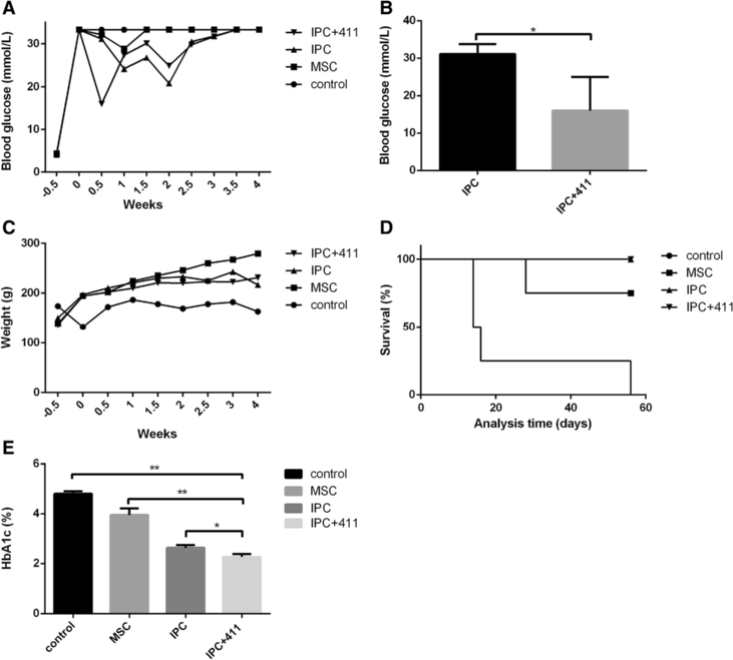
**Blood glucose level, weight and survival analysis of STZ induced T1DM rats. (A)** Fasting blood glucose levels were monitored twice a week as indicated in samples obtained from the tail vein using Blood Glucose Test Strips (Roche Diagnostics, Basel, Switzerland). Blood glucose over 33.3 mmol/L was beyond the measuring scale. **(B)** Fasting blood glucose level 3 days after IPCs with laminin 411 infusion, Bars: mean ± SEM (n = 3), and *P < 0.05. **(C)** Weights were monitored twice a week as indicated. **(D)** Survival curve of animal tests. **(E)** HbA1c level at the endpoint of the experiments. Bars: mean ± SEM (n = 4), and *P < 0.05, **P < 0.01.

To evaluate the therapeutic effect of the IPCs, T1DM animal models were generated as described in the materials and methods. FBG was monitored to assess glycaemia (Figure 8A). On the 3^rd^ day of cell transfusion, FBG level was significantly lower than IPC and MSC groups, respectively (*P <* 0.05, IPC + 411 vs IPC, Figure 8B). No significant difference in FBG levels between IPC and MSC groups vs control, respectively, was found (IPC vs control; MSC vs control, data not shown). However, the FBG level increased 2.5 weeks after cell infusion. Afterwards, the FBG level of all groups reached up to 33.3 mmol/L, which was beyond the detectable range of the glucose trips. We also observed that diabetes symptoms such as polyuria and polydipsia of T1DM rats were more effectively ameliorated by IPC + 411 transfusion than by IPC or MSC. In addition, the weights of the rats that received IPC + 411, IPC or MSC transfusion were slightly increased compared with controls (Figure 8C). Both IPC + 411 and IPC transfusion groups showed elongated survival time than MSC group (Figure 8D). IVGTT test was done at day 50 of the animal experiment, but no significant differences in GTT between IPC + 411 and IPC were observed, indicating that transfused IPC did not correct intrinsic β cell activity and treatment efficacy was primarily attributable to insulin secreted by the IPCs. Up to 70 days of the cell transfusion, the survival rate in IPC + 411 and IPC group was 100%, while MSC group 75% and the control group 0%.

Our results demonstrated that the HbA1c concentration in the blood of the rats treated by IPC + 411 was significantly decreased when compared with that of control, IPC and MSC groups, respectively, as shown in Figure 8E (*P <* 0.01, IPC + 411 vs control; *P <* 0.05, IPC + 411 vs IPC; IPC + 411 vs MSC; *P <* 0.05, respectively). (In these comparisons made, newly generated T1DM models from the same rat strain by following the same protocol were applied as controls). Meanwhile, the difference in HbA1c concentration between IPC and control, and MSC and control groups reached significance, respectively (*P <* 0.05, IPC vs control; *P <* 0.05, MSC vs control). These results suggested that therapeutic effect of IPC + 411 on T1DM was much more efficient than IPC and MSC.

## Discussion

In this study, we reported a laminin 411-containing protocol to induce UC-MSC into IPCs. Based on our present findings, combined with published studies, we believe that the protocol reported here has the following advantages: First, the IPC generation is free from gene manipulation, which guarantees safety in the future clinical application. Second, laminin 411 more effectively increases the yield of IPCs and insulin secretion. Third, the administration of laminin 411-exposed IPCs significantly attenuates FBG, HbA1c and improves the survival in DM rats, which demonstrates their therapeutic efficacy *in vivo*.

IPCs was first described to be generated from mouse ESCs [18]. Thereafter, generation of IPCs from human neural progenitor cell [22], ESCs [23], liver cells [24,25], pancreatic non-endocrine cells [26], placenta-derived multipotent stem cells [27], gene modified BM-MSC [5], and pancreatic cells [28] have been reported. With respect to efficiency of IPC differentiation and maturation, protocols used in the previous studies have certain disadvantages. First, the induction duration of IPCs is always longer than 21 days or even more than 2 months. Second, only about 20% of these IPCs could secrete insulin [22]. Third, insulin release of IPCs was still insufficiency *in vitro*[29]. As shown in the present study, using our laminin 411-containing protocol, only about 2 weeks were required for IPC generation from UC-MSCs and up to 612.1 ± 40.78 μIU/ml insulin release *in vitro*.

MSCs appear to be promising stem cells applied in clinical setting. MSCs were first described in BM (BM-MSC). However, there is age-related differences in the differentiation capacity of BM-MSCs and AD-MSCs [30-32], donor age and long term passages also affect the characteristic of BM-MSC [33]. To apply UC-MSCs have several advantages. Firstly, umbilical cord tissues are easy to obtain without invasion and are medical wastes during a natural delivery. Secondly, UC-MSCs have less ethical problems than ESCs. Thirdly, UC-MSCs could be large-scale expansion *in vitro* under GMP condition. Moreover, evidence shows that proliferation ability and pluripotency markers of UC-MSCs are better than those derived from BM [34].

Laminin 411 was one kind of extracellular matrix (ECM) protein. ECM contains fibronectin, laminin, and collagens, which can profoundly influence stem cell fate choices [35]. Laminin was found to support mouse ESCs proliferation in vitro [36] and soluble laminin could increase insulin secretion by 20% in murine islets *in vitro*[37]. In islet transplantation, treating islets with laminin prior to transplantation will help to maintain insulin production until new capillaries are formed in transplanted islets. Murine insulinoma cell line MINI6 cultured in laminin 411 could increase the insulin gene expression significantly [38]. Lin observed that fibronectin and laminin could induce the *hTERT-over-expression* BM-MSC into IPCs [11]. However, the type of laminin used in their study was unclear. Our findings clearly demonstrated that laminin 411 was the one capable of inducing maturation and differentiation of IPCs. Flanagan reported that laminin 411 might be the ligand for MCAM (CD146) and CD29 [39]. CD146 and CD29 are shown to be expressed in MSCs [40-44]. Ligation of the receptor(s) with laminin 411 on MSCs may regulate IPC differentiation and maturation.

Our results revealed that laminin 411 could induce *Pdx*1 and *Ngn*3 expression both at mRNA and protein levels, which provides a clue of how laminin 411 increases the maturation of IPCs. *Pdx1* also known as *IPF-1*, is the master regulator of the pancreatic development, and plays a crucial role during the development and function of pancreatic β-cells [45]. Mice and humans lacking *Pdx1* are apancreatic in the embryonic development [46]. *Pdx1* transfected into human BM-MSC could induce IPCs and injected to diabetes mice could sustain a decline of blood glucose more than 80 d [9]. During the ontogeny of pancreatic β-cells, pancreatic progenitor cells that express *Ngn3* would develop into endocrine cells. In those cells, only *Pdx1*^+^*Ngn3*^+^ progenitor cells could develop into insulin-producing β-cells [47]. Mature pancreatic β-cells are also *Pdx1* positive. Our current protocols of IPCs differentiation markedly induced *Pdx1*^+^ expression, indicating that laminin 411 may play essential roles in regulation of the yield of IPCs. Moreover, the presence of laminin 411 could markedly increase the IPC cluster number and size. Of note, the laminin 411 exposed-cells harvested in the middle producer of IPC induction were also shown to express high level of *Pdx1*, indicating that laminin 411 may induce MSC differentiation into pancreatic progenitor cells (intermediate insulin producing cells) first prior to IPC formation.

Definitive Endoderm (DE) is defined as a population of squamous cells that expressed *Foxa2*, *Sox17* and *CXCR4*, which is formed after the prior formation of primitive endoderm [48]. Only DE can be further differentiated into specific endoderm lineages [21]. Therefore, we determined whether laminin 411 could regulate the expression of DE. Our results showed that laminin 411 markedly increased the expression of *Sox17* and *Foxa2*. Furthermore, insulin secretion level was positively correlated with *Foxa2* and *Pdx1* expression. Therefore, our results demonstrate that laminin 411-induced differentiations of MSCs into IPC may be through the DE pathway.

In the cell transfusion experiments with DM rats, IPC + 411 group decreased the FBG level significantly at the 3rd day after transfusion, and much earlier than IPC and MSC groups. Afterwards, FBG level of the rats treated by IPC + 411 was gradually increased and reached to 33.3 mmol/L after two and half weeks. Of note, 33.3 mmol/L (600 mg/dL) is the highest level that could be measured by the technique glucose trips applied in current study. Due to such technical limitation, we did not know exact glucose levels of rats between different groups after the cell transfusions beyond two and half weeks. Nevertheless, we found that rats treated with IPC + 411 presented less DM symptoms than the IPC group, indicating a better blood glucose control by IPC + 411 than IPC. In line with this speculation, our results of HbA1c analysis demonstrated that at the end of the experiment (on day 70 after the cell transplantation) the HbA1c level in IPC + 411 treatment group was significantly lower than control and other cell transfusion groups. Considering the facts that HbA1c was a maker reflecting the average blood glucose level over the previous 2 to 3 months and higher level of HbA1c indicates poorer control of blood glucose levels, our findings above suggest a long term therapeutic or blood glucose control effect of IPC + 411 on T1DM. We also observed that IPC + 411 showed more superior long-term therapeutic effects over the IPC without exposure to laminin 411 and MSC in terms of survival and disease symptom control.

## Conclusion

Our laminin 411 containing IPC induction protocol significantly enhanced differentiation efficiency of IPCs from MSCs derived from human cord tissues. Moreover, transfusion of the IPCs generated from laminin 411 protocol resulted in a long-term therapeutic effects on T1DM. Our findings here highlight a crucial role of laminin 411 in the induction of IPCs differentiation from human MSC and a potential clinical application of the IPCs + 411 in the treatment of T1DM as well as type 2 DM in the future.

## Abbreviations

DM: Diabetes mellitus; MSC: Mesenchymal stem cells; BM: Bone marrow; UC: Umbilical cord; AD-MSC: Adipose derived mesenchymal stem cell; FBS: Fetal bovine serum; DMSO: Dimethylsulfoxide; ELISA: Enzyme-linked immunosorbent assay; PBS: Phosphate buffered saline; PFA: Paraformaldehyde; IPC: Insulin producing cells; STZ: Streptozotocin; ECM: Extracellur matrix; Pdx1: Pancreatic and duodenal homeobox gene 1; DE: Definitive endoderm; RT-PCR: Real time polymerase chain reaction.

## Competing interests

The authors declare that they have no competing interests.

## Authors’ contributions

HTQ carried out experiments, analysed and interpreted data and drafted the manuscript. CYZ, DQW and DWX contributed to study design; CYZ contributed to technique guidance, data interpretation, and constructing and revising the manuscript; DWX contributed to revising manuscript. XLL, XLF, YHN, YJ, XML, DXK, JDW, ZLW, YJW, XHZ, and SHC contributed to technical assistance, experiment involvement, statistical analyses and manuscript revision. All authors read and approved the final manuscript.
